# Gambling in a U.S. Census Matched Sample: Examining Interactions between Means and Motives in Predicting Problematic Outcomes

**DOI:** 10.1007/s10899-024-10302-w

**Published:** 2024-04-09

**Authors:** Christopher G. Floyd, Shane W. Kraus, Joshua B. Grubbs

**Affiliations:** 1 Department of Psychology, Bowling Green State University, 822 E. Merry Ave, Bowling Green, OH 43403 USA; 2 Department of Psychology, University of Nevada Las Vegas, 4505 S. Maryland Parkway, Las Vegas, NV CEB 320, 89154 USA; 3 Center on Alcohol, Substance Use, And Addictions (CASAA), University of New Mexico, 2650 Yale BLVD SE, Albuquerque, NM USA

**Keywords:** Financial Gambling Motives, Socioeconomic Status, Problem Gambling, Perceived Deprivation

## Abstract

The influence of socioeconomic status (SES) on risk of Problem Gambling (PG) is complex, particularly given recent evidence that SES should be understood in both objective and subjective terms. Likewise, financial gambling motives have been found to be predictive of PG; however, financial motives are less understood in comparison to other gambling motives. Preliminary findings on SES and gambling points towards a pattern of social inequality in which those with the least financial resources (e.g., income) or that feel financially deprived relative to others (e.g., perceived deprivation) experience greater harm and problems. In a weighted, census matched sample of adults in the U.S. (*N* = 1,348), the present study examined the interaction between financial gambling motives and income and financial gambling motives and perceived deprivation in predicting PG. Findings provided support for both financial gambling motives and perceived deprivation as robust predictors of PG. Further, results provided unique insights into the role subjective economic standing may play in the relationship between financial motives for gambling and risk of PG.

The landscape of gambling in the United States (U.S.) has changed significantly in recent years, with increased legalization of gambling throughout the U.S. and internet technology enabling access to gambling at unprecedented rates (Lawn et al., 2020; Winters & Derevensky, 2019). Such increases in gambling access have led to increasing concerns about gambling as a potential threat to public health (Latvala et al., 2019). Specifically, the exploding popularity of the online gambling market has led to concerns regarding the rising rates of gambling participation (Sirola et al., 2018), suggesting the possibility of more widespread gambling-related harms (Díaz & Pérez, 2021; Newall, 2023; Mora-Salgueiro et al., 2021). In some ways this rise in attention to gambling has led to increases in gambling research (Abbott, 2020), although there are numerous domains in which gambling behaviors and associated problems remain poorly understood by scholars, policy makers, and gaming regulators. One such domain is the links between socioeconomic status (SES) and psychological motives underlying gambling disorder.

While it is well-known that social and economic circumstances influence risky behaviors, there is still much unknown about the socioeconomic roots of problem gambling (Van der Maas, 2016; Welte et al., 2004a). There is similarly little work seeking to understand how SES and perceived economic standing may interact with gambling motives to predict problematic gambling-related outcomes. Examining these associations appears prudent given that gambling motives (Estévez et al., 2021) and SES (Canale et al., 2017; Richard et al., 2017) have both been shown to be predictors of problem gambling. As such, the present work examines the interaction between gambling motives—particularly financial motives— and SES in predicting problem gambling behaviors. Additionally, the present study explored whether perceived relative deprivation influences the proposed effect of financial gambling motives on gambling severity, with a goal of identifying risk factors for problem gambling behavior, particularly among economically and socially disadvantaged populations who are disproportionately impacted (Abbott, 2020; Sharman et al., 2019).

## Gambling and Problem Gambling

Although gambling is a common, socially acceptable recreational activity that is harmless for most people, for a small minority of people, gambling can become problematic (Meyer et al., 2009), culminating in an addictive disorder known as Gambling Disorder ([GD]; Yau & Potenza, 2015). The American Psychiatric Association’s *Diagnostic and Statistical Manual of Mental Disorders*-*Fifth Edition* (DSM-5; APA, 2013) defines GD as ‘persistent and recurrent problematic gambling behavior leading to clinically significant impairment or distress’ and articulates that it is characterized by preoccupation, escalation, and failed attempts at regulating or restricting gambling-related behavior despite significant distress or consequences (APA, 2013; Potenza et al., 2019). However, gambling can also be problematic at subclinical levels, which is termed problem gambling (abbreviated: PG). PG has been found to affect up to 4.6% of the adult population in the U.S. and is associated with various gambling-related harms (Welte et al., 2015).

### Gambling and Financial Harm

Financial distress is potentially the most common gambling-related harm (Browne et al., 2016; Langham et al., 2015). Among a clinical sample of help-seeking problem gamblers, 87% of the sample reported gambling-related financial harms and approximately half (45%) of the sample reported being in a vicious cycle of debt (Salonen et al., 2018). Furthermore, the financial harms associated with gambling are often the impetuses of or mediating factors in many other gambling-related harms, such as relational struggles (Hing et al., 2013; Jeffrey et al., 2019) and psychological gambling-related harms (Oksanen et al., 2018; Swanton & Gainsbury, 2020).

When considering the significance of financial related harms in the spectrum of PG, it stands to reason that financial harms cannot be easily disentangled from SES. More directly, the association between PG and harms may be conditioned on a predictable pattern of social inequality in which those with the least socioeconomic resources experience greater financial harms associated with their gambling behaviors (Raybould et al., 2021; Latvala et al., 2021).

### Socioeconomic Status and Gambling Behavior

The American Psychological Association defines socioeconomic status (SES) as “the social standing or class of an individual…” and states that it is often measured as a combination of education, income, and employment (APA, 2016). Despite the substantial influence SES has been shown to have on social psychological processes, it remains insufficiently considered in psychological literature. Even so, psychological research on SES has grown significantly in recent years, and the need to integrate SES and perceptions of social class in psychological scholarship has become apparent (Diemer et al., 2013).

There is clear evidence that SES should be understood both in absolute terms (i.e., objective measures of income, education, employment) and subjective terms. That is, how people perceive their own SES often shapes how they interact with the world (Destin et al., 2017; Manstead, 2018) and seemingly affects how SES itself influences outcomes (Diemer et al., 2013). Accordingly, there has been an increase in interest in subjective social status, which refers to one’s perception of their social class in relation to others (Demakakos et al., 2008). Though inherently less objective than measures of income, subjective social status is often a key factor in understanding how things like income influence relevant psychological outcomes.

While research has been conducted on the impact of SES, particularly income (Williams et al., 2023), on gambling behavior and outcomes, there is a need for research that integrates SES and psychological principles associated with gambling (e.g., motives and cognitions). The preponderance of PG studies has focused on individual attributes associated with risk and often lack a focus on social and environmental factors of gambling, such as SES. As such, there is a dire need for research that examines how larger trends in social disadvantage might shape gambling behavior and outcomes (Richard et al., 2017).

The above observations notwithstanding, some work does demonstrate the influence of SES on gambling outcomes. For example, individuals who report lower SES experience greater gambling-related harms than more affluent individuals (Krisnanda et al., 2023; Raybould et al., 2021). Specifically, gambling-related harms and risks are associated with lower income, lower education, and unemployment (Hahmann et al., 2021; Hagfors et al., 2023). Income may be a particularly important SES factor for consideration in research on GD, as it appears that those with lower incomes are at increased odds of reporting a lifetime history of GD or PG (Day et al., 2020).

### Disadvantage at the Individual and Community Level

It is unlikely that those in a higher income bracket experience the same monetary and social consequences of gambling as those living in or near poverty. The possibility exists that the economic costs of gambling disproportionally fall on the shoulders of those who lack the resources to manage the monetary and social losses associated with gambling. Likewise, PG prevalence rates have been found to be higher among those experiencing poverty (Hahmann & Matheson, 2017). Additionally, people with lower incomes, on government assistance, and without a job are at increased risk of experiencing gambling problems (Hahmann et al., 2021). In addition to individual economic disadvantage, research has examined how neighborhood disadvantage impacts gambling behavior and outcomes. Using a U.S. representative sample, Barnes et al. (2013) found that those living in the poorest neighborhoods had greater odds of developing PG (Barnes et al., 2013). The association between low SES and PG can be explained, in part, by the higher concentrations of gambling opportunities in economically disadvantaged neighborhoods and communities (Doran & Young, 2010; Rintoul et al., 2013). Given that disadvantage at the individual and neighborhood level may increase one’s risk for PG or GD, future research on the relationship between SES and PG is warranted.

In sum, low SES, in each of its aspects (e.g., income, education, and employment), is associated with more gambling-related harms (Hahmann et al., 2021; Petry, 2005; Welte et al., 2004a) and problems (Barnes et al., 2013; Day et al., 2020). In addition, low-income groups, such as those living in poverty, have been shown to gamble a greater proportion of their income despite lacking the resources to manage the monetary consequences (Hahmann et al., 2021). Lastly, economic disadvantage at the individual and neighborhood level increases risk of gambling-related harm and problems, which is exacerbated by the evidence that gambling opportunities are substantially higher in economically disadvantaged neighborhoods and communities (Doran & Young, 2010; Rintoul et al., 2013). This review of this literature leads to two clear conclusions: (1) SES appears to be related to gambling behavior and outcomes (Carbonneau et al., 2015), and (2) the current gambling literature, due to a historical lack of consideration of SES, fails to provide sufficient knowledge on the topic.

### Perceived Deprivation and Gambling

Perceived deprivation refers to the belief that one is worse off financially compared to others and does not have what is deserved (Smith et al., 2012). In short, it is how one feels about their economic standing in relation to others. Callan et al. (2008) hypothesized that gambling may be an alternative, less conventional way to behaviorally compensate for perceived deprivation and demonstrated that participants that felt relatively deprived were more likely to gamble (Callan et al., 2008). Evidence suggesting desire to gamble increases with perceived deprivation has been demonstrated in subsequent works (Callan et al., 2011; Haisley et al., 2008).

Perceived deprivation, particularly among those of lower income (Wilkinson & Pickett, 2009) may motivate an individual’s desire for upward economic mobility, resulting in engagement in high-risk behavior to reduce financial disadvantage (Callan et al., 2015). Research has also demonstrated a positive association between having a self-concept that is overly focused on financial success (i.e., financially focused self-concept) and perceived deprivation, potentially due to financially focused people being preoccupied with comparing the amount of money they have with the amount other people have (Tabri et al., 2017a). Furthermore, using a community sample of gamblers, Tabri et al. (2017b) tested a moderated mediation model in which the effect of perceived deprivation on disordered gambling is moderated by financially focused self-concept. Findings demonstrated that perceived deprivation predicts disordered gambling severity, particularly among gamblers with a financially focused self-concept (Tabri et al., 2017b).

Building on the above, gambling among lower-income individuals may function as a form of justice-seeking due to gambling being perceived as a means of attaining desired outcomes that people feel are deserved but are unable to attain via conventional means (Callan et al., 2008). That is, people may gamble for financial reasons due to the belief that upward economic mobility is unattainable through conventional pursuits. Furthermore, meta-analytic results of the links between perceived deprivation and gambling behaviors indicate that perceived relative deprivation is associated with stronger urges to gamble (*r* = .26) and that, across studies, the strength of the relationship is moderated by PG severity (Callan et al., 2015). Collectively, these findings suggest that there might be an effect of perceived deprivation on the motivations people have for gambling.

### Gambling Motives

Motives underlying gambling behaviors have been extensively studied in recent decades (Dechant, 2014). Not surprisingly, among the many motives a person might describe as underlying their gambling behaviors, financial motives are likely to be a consistent theme. Indeed, winning money is often the most endorsed item response to survey data asking gamblers about their motives for gambling (McGrath et al., 2018; Neighbors et al., 2002). Despite this, financial motives for gambling are among the most overlooked and understudied explanations for why people gamble (Tabri et al., 2022).

The lesser focus on financial gambling motives is noteworthy given the research suggesting that financial motives are common among problem gamblers (Dechant, 2014; Pilatti & Tuzinkievich, 2015). Research on gambling motives has consistently found that the inclusion of financial motives improves the prediction of gambling frequency and PG (Dechant, 2014; Lee et al., 2007; Pilatti & Tuzinkievich, 2015) in both clinical and community samples (Devos et al., 2017; Schellenberg et al., 2016; Tabri et al., 2022). Furthermore, assessing financial motives has provided unique insights into characteristics and preferences that distinguish gamblers who endorse financial motives for gambling (Barrault et al., 2019; Van der Maas et al., 2019).

Despite the above findings illustrating the importance of assessing financial motives for gambling, some findings regarding financial motives are quite mixed. Some research indicates that financial motives may predict gambling frequency but not the development of PG (Barrada et al., 2019). Alternatively, other studies have found a relationship between greater financial motives and greater problems when controlling for level of gambling involvement (Marmurek et al., 2014). Consistent with the common finding that PG is mainly maintained by mood enhancement and coping motives (Flack & Morris, 2015; Marchica et al., 2020; Wood & Griffiths, 2007), studies report that a positive bivariate association between financial motives and PG is attenuated when controlling for shared variance with other motives (Flack & Stevens, 2019; Kim et al., 2019). Still, modern works have demonstrated a positive association between financial motives and PG after controlling for other motives (Barrault et al., 2019; Tabri et al., 2022). While these inconsistent findings could be explained by financial motives being a less robust predictor of PG in comparison to emotion-focused motives, larger limitations with the research, such as psychometric issues with the measurement of financial motives, are also plausible (see Tabri et al., 2022). Even so, the inconsistent links between financial motives for gambling and symptoms of PG and GD are cause for further research, suggesting that other variables might be influencing the relationship between financial motives and symptoms of PG/GD.

#### Financial Motives and Perceived Deprivation

Some research exists examining the relationship between perceived relative deprivation and financial motives for gambling. For example, Tabri et al. (2015) found evidence that the effect of perceived deprivation on disordered gambling severity was mediated by financial gambling motives. Findings also demonstrated that this effect was moderated by perceived upward economic mobility (Tabri et al., 2015). That is, findings suggested that the effect of relative deprivation on financial motives to gamble may be stronger among people who perceive that they lack the capacity for economic mobility through more conventional means. In summation, research suggest that holding a financially focused self-concept (Tabri & Wohl, 2021) and perceptions of being financially deprived relative to others (Callan et al., 2008; Tabri et al., 2017b) are associated with financial gambling motives and having gambling problems.

## Present Study

The discussed research underscores the need to consider the potential impact that SES differences can have in shaping gambling motivations and behavior, and thus problematic outcomes of gambling (e.g., PG and GD). To address these gaps in the literature, the present study includes a test of the interaction between financial motives for gambling and income in predicting problem gambling behaviors and outcomes. In addition, the present study explored whether perceived deprivation influences the proposed effect of financial gambling motives on gambling severity.

The present study has three primary aims: (1) examine if financial gambling motives are predictive of PG severity, (2) examine whether financial gambling motives and income interact to predict PG severity, and (3) explore whether the relationship between financial gambling motives and PG severity varies based on feelings of being financially deprived (i.e., perceived relative deprivation). The aforementioned aims are based on prior works demonstrating an association between financial motives for gambling, perceived relative deprivation, and adverse outcomes of gambling (Callan et al., 2015; Tabri et al., 2015, 2017b, 2022).

Building on the above literature, we expected that self-reported financial gambling motives would have a significant positive, direct effect on PG severity (H1). Second, we expected to find that the interaction of self-reported financial gambling motives and income would transmit a significant negative effect on PG severity. Specifically, it was hypothesized that the effect of financial gambling motives on PG severity would be strongest at lower (relative to higher) levels of income (H2). Third, we expected that perceived relative deprivation would have a significant positive, direct effect on PG, so that higher levels of perceived relative deprivation are associated with increased PG severity (H3). Lastly, we expected that the interaction of self-reported financial gambling motives and perceived relative deprivation would transmit a significant positive effect on PG severity. Specifically, it was hypothesized that the positive predictive effect of financial motives on PG severity would be strongest at higher (relative to lower) levels of perceived relative deprivation (H4). Conceptual models of these hypotheses are available at: https://osf.io/9xbv8/.

## Method

### Participants and Procedure

Participants were recruited by YouGov Opinion Polling as a part of a larger study (see Grubbs & Kraus, 2022). In March 2022, a sample was recruited via YouGov America of American adults (*n* = 2,806, *M*_*age*_*[SD] =* 48.9 [17.2]; 1365[48.6%] men; response rate = 87.6%), matched to U.S. norms for age, gender, education, census region, and race/ethnicity as of the 2019 American Community Survey (ACS; US Census Bureau, n.d.).

YouGov uses a sample-matching method to construct census-matched samples from a large online opt-in panel. For the present data, YouGov drew a random hypothetical sample based on the full American Community Survey which corresponds to the sampling frame and selects panelists who match the characteristics of the sampling frame member. YouGov provided poststratification weights, based on age, race ethnicity, educational level, and both 2020 and 2016 vote history, when available; however, such weights were not used in the present analyses given that the weights were based on the total sample and not the subset of the sample used in the present study. As the data was obtained by YouGov Opinion Polling, specific measures of quality control and responsiveness were guaranteed as a part of their data collection process and based on their proprietary data collection standards. In the present data, the survey routing ensured that gambling measures were completed only by those who endorsed past year gambling of any form (*N* = 1,348). The median income for the final sample ranged from $50,000 to $59,000 (*M* = 6.43; *SD* = 3.449). Full demographics for the present studies sample are available in Table 1. It is worth noting that the use of an online opt-in panel (i.e., YouGov) may introduce sampling bias, limiting the generalizability of findings. However, YouGov has demonstrated its utility as a means of studying gambling at the population level (Wardle & McManus, 2021). While YouGov can overestimate rates of PG, and thus should not be used to make epidemiological conclusions, it has been found to be useful for understanding gambling behavior in the population more broadly (Sturgis & Kuha, 2021).

**Table 1:** Demographics

Variable	N	%
Gender		
Female	642	51.5%
Male	694	47.6%
Non-binary	7	0.5%
Other	5	0.4%
Age	55.00 (*SD* = 15.49)	
Ethnicity		
White	910	67.5%
Black	166	12.3%
Hispanic	181	13.4%
Asian	26	1.9%
Native American	14	1.0%
Two or more races	24	1.8%
Other	25	1.9%
Middle Eastern	2	0.1%
Marital Status		
Married	626	46.4%
Separated	29	2.2%
Divorced	190	14.1%
Widowed	76	5.6%
Never married	324	24.0%
Domestic/civil partnership	103	7.6%
Education		
No HS	49	3.6%
High school graduate	449	33.3%
Some college	269	20.0%
2-year	143	10.6%
4-year	277	20.5%
Post-grad	161	11.9%
Employment Status		
Full-time	542	40.2%
Part-time	132	9.8%
Temporarily laid off	10	0.7%
Unemployed	79	5.9%
Retired	337	25.0%
Permanently disabled	127	9.4%
Homemaker	74	5.5%
Student	26	1.9%
Other	21	1.6%
Median Family Annual Income	$50,000-$59,999	

### Measures

#### Income

The present study measured annual income, an objective SES indicator, using a single item from the demographics portion of the survey which asked participants to indicate their annual income on a range from 1 (less than $10,000) to 16 ($500,000 or more). A full list of the income categories is available at https://osf.io/9xbv8/.

#### Personal Relative Deprivation

To measure perceived relative deprivation, an abbreviated version of Callan et al.’s (2008) Personal Relative Deprivation Scale (PRDS) was used. The original PRDS is a 4-item questionnaire that was designed to assess individuals’ self-reports of their personal relative deprivation and feelings of resentful about what they have compared to what other people have. Consistent with past findings (Callan et al., 2008), a reliability analysis indicated that the 4-item PRDS did not have good internal consistency (*α* = 0.645) when including the reversed scored items (2 and 4). In line with recommendations from Kim et al. (2018a), the two reversed scored items were dropped, resulting in 2-item PRDS with acceptable internal constancy (*α* = 0.76). Participants responded to the items (e.g., “When I think about what I have compared to others, I feel deprived” and “I feel resentful when I see how prosperous other people seem to be”) on a scale ranging from 1 (*strongly disagree*) to 6 (*strongly agree*).

#### Gambling Outcomes

Gambling outcomes was measured both in terms of frequency and problems, as measured by the Problem Gambling Severity Index (PGSI; Ferris & Wynne, 2001). The PGSI is a nine-item inventory that measures symptoms of PG/GD (e.g., “Have you bet more than you could really afford to lose?”; “Have you felt guilty about the way you gamble or what happens when you gamble?”) on a scale from 0 (*never*) to 3 (*almost always*).

#### Gambling Motives

Motives for gambling was measured using the Gambling Motives Questionnaire-Financial (GMQ-F; Dechant, 2014). The GMQ-F is a sixteen-item inventory in which participants rate the frequency of different gambling motives using a quasi-interval four-point scale (1 = *never or almost never*; 2 = *sometimes*; 3 = *often*; 4 = *almost always or always*). The GMQ-F includes four subscales: Enhancement, Coping, Social, and Financial Motives. For the purposes of the present study, only the Financial Motives subscale was used. The Financial Motives subscale includes 4-items that measure financial reasons for gambling (e.g., “To win money”). A full list of the items for each measure used in the present study is available at: https://osf.io/9xbv8/.

### Analytic Plan

The Data was provided by YouGov in SPSS format. The analyses were conducted in SPSS. In addition, the focal predictor (financial gambling motives) and the moderators (income and perceived relative deprivation) were centered prior to conducting analyses. Descriptive statistics for included measures were calculated (See Table 2). Bivariate correlations, as well as measures of internal consistency (Cronbach’s alpha and Omega total), were reported across all included measures and reported prior to the primary analyses (See Table 3). Effect sizes were interpreted in light of recent benchmarks (e.g., correlations: < 0.10 are very small; < 0.20 are small; <. 30 are moderate, < 0.40 are large, and > 0.40 are very large; Funder & Ozer, 2019).

To the study aims, OLS regression moderation analyses were conducted to examine if the variables of interest explained a significant amount of variance in the study’s dependent variable (i.e., PG). First, income was tested as a moderator in the relationship between financial gambling motives and PG severity, then perceived deprivation was tested as a moderator in the relationship between financial gambling motives and PG severity. The moderation analyses were conducted, and the interaction results were plotted using the InterActive application (McCabe et al., 2018). This open-source, web-based tool allows for moderation analyses using OLS techniques, while producing more visually intuitive graphs of results. Annual income and perceived deprivation were tested separately as the moderators in the relationship between financial gambling motives and PG severity. In total, two moderation analyses (one for income and one for perceived deprivation) were plotted.

**Table 2:** Descriptive statistics for key variables

Variable	Range	M (SD)	Skew	Kurtosis	SE
Financial Gambling Motives	1–4	2.298 (0.843)	0.330	− 0.874	0.023
Problem Gambling Severity	0–3	0.255 (0.528)	2.783	7.796	0.014
Income	1–16	6.430 (3.449)	0.293	− 0.837	0.094
Perceived Deprivation	1–7	2.984 (1.517)	0.452	− 0.618	0.041

## Results

In general, the correlations between the present study’s variables were consistent with expectations, with a few exceptions. As expected, a positive moderate correlation between financial gambling motives and PG severity was found (*r* = .315, *p* < .001). Further, the correlation between financial gambling motives and income was negative and small in magnitude (*r* = − .149, *p* < .001). The correlation between financial gambling motives and perceived deprivation was positive and small in magnitude (*r* = .205, *p* < .001). Expectedly, a small negative correlation was found between income and PG severity (*r* = − .117, *p* < .001). Consistent with expectations, a positive moderate correlation between perceived deprivation and PG severity was found (*r* = .340, *p* < .001). Lastly, a small negative correlation was found between income and perceived deprivation (*r* = − .133, *p* < .001).

**Table 3:** Pearson’s r, means, and standard deviations

Variable	1	2	3	4
(1) (1) Financial Gambling Motives	1			
(2) Problem Gambling Severity	0.315^**^	1		
(3) Income	− 0.149^**^	− 0.117^**^	1	
(4) Perceived Deprivation	0.205^**^	0.340^**^	− 0.133^**^	1
Cronbach’s α	0.80	0.95	NA	0.76
McDonald’s ω	0.80	0.95	NA	NA

*Correlation is significant at the 0.05 level (2−tailed)

**Correlation is significant at the 0.01 level (2−tailed)

### Results of Moderation Analyses

**Fig. 1:**
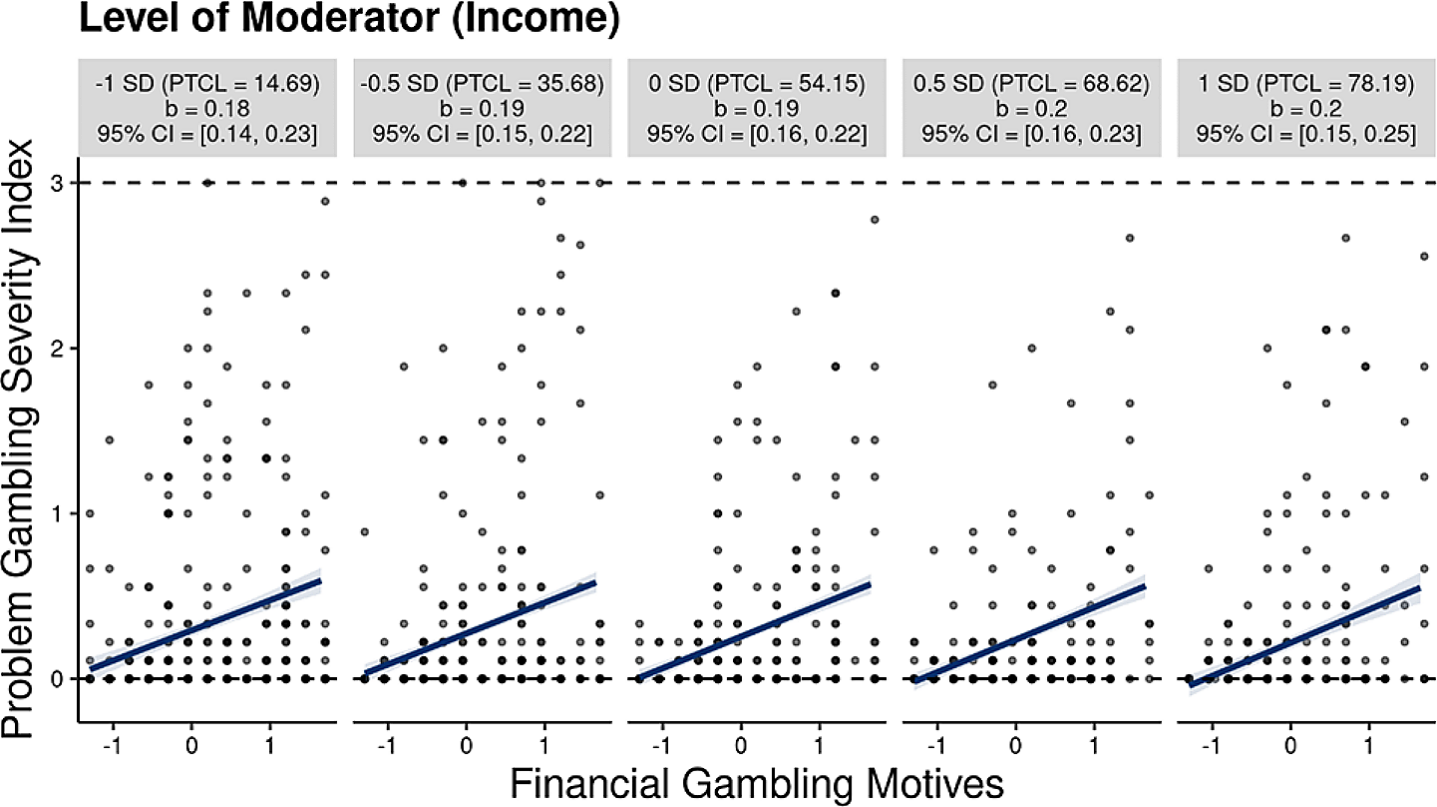
Visualization for moderation analysis for income

As stated, annual income and perceived deprivation were tested separately as moderators in the relationship between financial gambling motives and PG severity (see Tables 4 and 5 for full summary of the moderation analyses). For the moderation analyses with annual income as the moderator, OLS results revealed that financial gambling motives had a positive effect on PG severity (*b* = 0.192, *p* = < 0.001, 95%, CI = [0.159, 0.224], *β* = 0.306). That is, findings demonstrated support for H1, indicating that higher levels of financial gambling motives were associated with increased PG severity. Further, income was found to have a negative effect on PG severity (*b* = − 0.011, *p* = .007, CI = [-0.019, -0.003], *β* = − 0.070). Findings did not demonstrate support for H2, as findings showed that the interaction of financial gambling motives and income did not have a significant effect on PG severity scores (*b* = 0.003; *p* = .565 CI = [-0.007, 0.012], *β* = 0.015). A visualization of the insignificant interaction (-0.1 *SD* to + 1 *SD*) is available in Fig. 1.

**Fig. 2:**
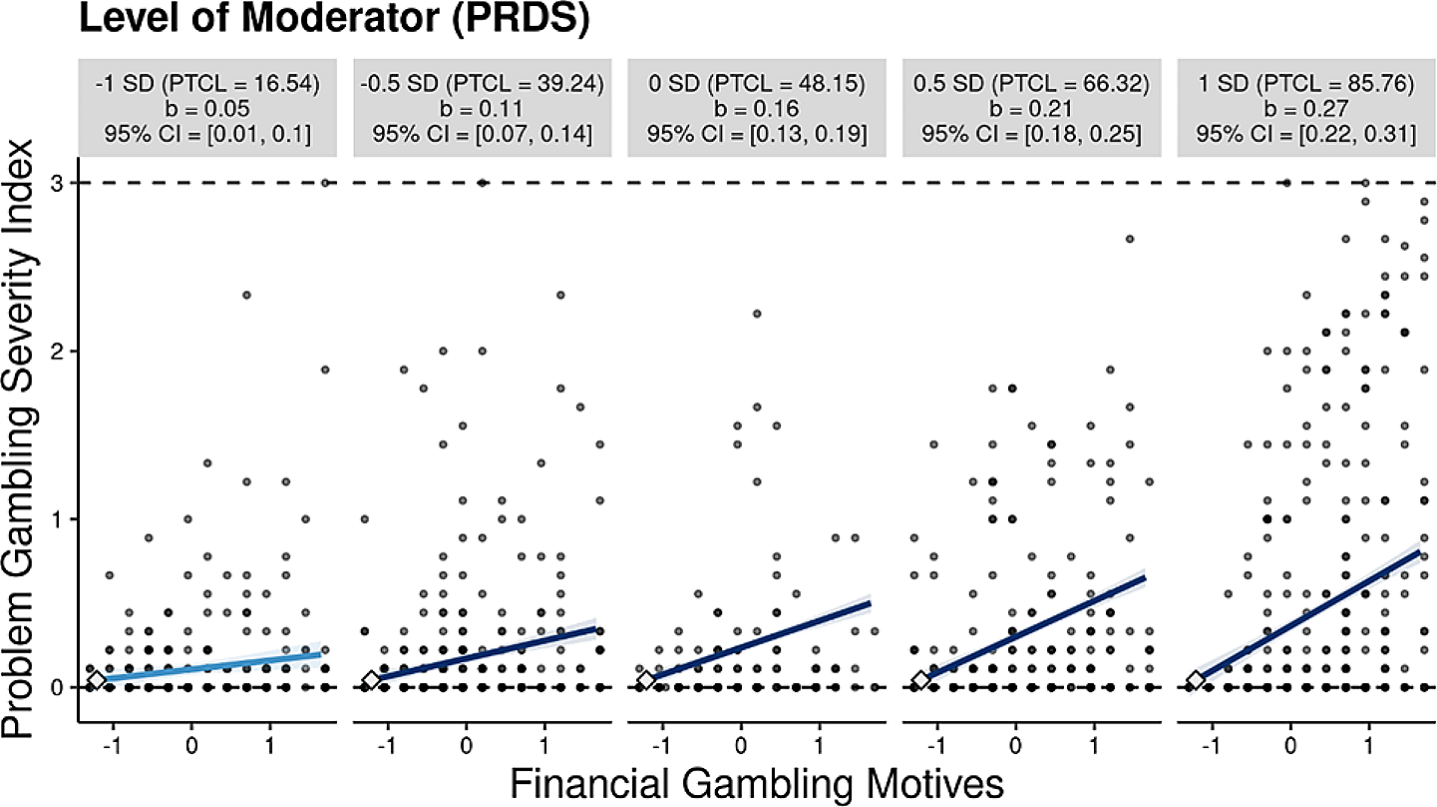
Visualization for moderation analysis for PRDS

For the moderation analyses with perceived deprivation as the moderator, findings supported H4. That is, findings showed that the interaction of financial gambling motives and perceived deprivation did transmit a positive effect on PG severity (*b* = 0.070, *p* = < 0.001, CI = [0.051, 0.090], *β* = 0.181). The moderation accounted for 3.1% of the variance in PG severity (*R*^*2*^ = 0.031, *F* = 52.668, *p* < .001). A visualization of the significant interaction (-1 *SD* to + 1 *SD*) is available in Fig. 2. As shown in Fig. 2, for participants low in perceived deprivation (i.e., -1 *SD*), every one unit increase in financial gambling motives was associated with a 0.05 increase in PGSI mean scores. However, for participants high in perceived deprivation (i.e., + 1 *SD*), a one unit increase in financial gambling motives was associated with a 0.27 increase in PGSI mean scores.

**Table 4:** OLS Results with income as the moderator

Variable	b	se	β	t	p	LLCI	ULCI
PGSI: *R* = .323, *R*^*2*^ = 0.104, *MSE =* 0.251, *F*(3, 1344) = 52.270, *p* < .001							
GMQ-F	0.192	0.016	0.306	11.668	< 0.001	0.159	0.224
Income	− 0.011	0.004	− 0.070	-2.689	0.007	− 0.019	− 0.003
GMQ-F x Income	0.003	0.005	0.015	0.575	0.565	− 0.007	0.012

**Table 5:** OLS Results with perceived deprivation as the moderator

Variable	b	se		β	t	*p*	LLCI	ULCI
	PGSI: *R* = .458, *R*^*2*^ = 0.209, *MSE =* 0.221, *F*(3, 1344) = 118.703, *p* < .001							
GMQ-F	0.160	0.016		0.255	10.293	< 0.001	0.129	0.190
PRDS	0.085	0.009		0.245	9.614	< 0.001	0.068	0.103
GMQ-F x PRDS	0.070	0.010		0.181	7.257	< 0.001	0.051	0.090

## Discussion

The present study aimed to extend upon prior works regarding the relationship between financial gambling motives and PG severity by examining whether the relationship varies based on annual income and perceived relative deprivation. Support was found for hypothesis 1, as a positive and moderately sized correlation between financial gambling motives and PG severity was found. However, findings did not support hypothesis 2, as the interaction of self-reported financial gambling motives and income was not found to transmit a significant effect on PG. Our third hypothesis was supported, demonstrating a positive moderate correlation between perceived deprivation and PG. We also found support for our fourth hypothesis demonstrating that the interaction of financial gambling motives and perceived deprivation had a significant positive small effect on PG. While findings provide added support for both financial gambling motives and perceived deprivation as robust predictors of PG, they also underscore the role subjective financial standing may play in the relationship between financial motives for gambling and risk of PG.

### Financial Gambling Motives and Problem Gambling

Since the development of the original Gambling Motives Questionnaire (GMQ; Stewart & Zack, 2008), gambling researchers have dedicated numerous studies to the motives underlying gambling behavior and demonstrated support for the GMQ’s three-factor solution. However, given the GMQ was not expanded to include financial motives until 2014, financial motives remain among the least studied explanations for why people gamble (Clarke et al., 2007; Dechant, 2014). Even so, a recent meta-analytic review of the association between financial gambling motives and PG confirmed that financial gambling motives are reliable and positive predictors of both gambling frequency and level of PG (Tabri et al., 2022). Results from the present work generally fall in line with these past findings, with financial motives being found to be a robust predictor of PG.

Tabri et al. (2022) also identified several limitations with the existing research on financial gambling motives, including a general lack of attention to perceived deprivation. A noted limitation of prior works on financial gambling motives and perceived deprivation has been the limited generalizability of results based on the sample source (Mishra & Meadows, 2018; Tabri et al., 2015). Consistent with recommendations in prior works for replicating findings using more representative samples (Tabri et al., 2015), the present study used a census-matched, stratified U.S. sample recruited via YouGov, which uses a sample-matching method that has been shown to outperform other probability and non-probability vendors (Kennedy et al., 2016; Rivers, 2016). As such, the present study’s findings are the first to examine these constructs in a census-matched, stratified U.S. sample.

### Socioeconomic Status and Gambling Behavior

As stated, the majority of studies on the relationship between SES and gambling have focused solely on objective indicators of SES (e.g., income, education, employment). Further, previous research has not substantively considered the role of SES in gambling problems among financially motivated gamblers. Moreover, the relationship between these constructs is not currently understood in a socioeconomic context (e.g., access to resources & sociocultural factors). Given the inherent monetary aspects of gambling, this study fills a gap in the literature regarding how larger trends in social disadvantage might shape gambling behavior and outcomes. That is, the present study did not merely examine the relationship between financially based motives for gambling and outcomes, it also examined the degree to which this relationship varies as a function of access to resources (i.e., income).

The present study demonstrated mixed results with regard to the relationship between financial gambling motives, income, and PG. While income, on its own, was found to be negatively correlated with PG, the interaction of financial gambling motives and income was not found to transmit a significant effect on PG. Given the dearth of research regarding whether the interaction of income and financial motives predicts PG, it is unclear why the interaction did not have a significant effect on PG severity. The non-significant interaction found in the present study could be a result of limitations associated with using household income, as opposed to personal disposable or discretionary income, which does not account for how resources are distributed among household members. Alternatively, this finding could be interpreted as indicating that objective indicators of SES, such as income, do not substantially influence whether financial motives increase the risk of gambling problems. However, caution is also warranted with regard this interpretation of findings given other national U.S. surveys indicating, while participation in most forms of gambling increases with SES, individuals of higher SES show lower rates of PG (Welte et al., 2002). This unexpected finding contrasts prior works that indicate being of lower income may increase risk of gambling problems (Day et al., 2020; Hahmann et al., 2021) and speculations that this association may be explained by financially based motives for gambling. For example, Welte et al. (2011), examined this in a nationally representative and weighted sample of adults (*N* = 2,631) and youth (*N* = 2,274) and demonstrated that gambling frequency and PG become more common as SES gets lower (Welte et al., 2011). Consistent with what was hypothesized in the present study, Welte et al. (2004b) speculated the association between SES and gambling problems may have something to do with lower SES Americans pursuing gambling as a means to make money (i.e., financial motives for gambling).

### Perceived Deprivation and Gambling Behavior

Consistent with the general push toward examining how not only objective SES but also subjective SES might impact individuals’ thoughts, motivations, and behavior (Adler et al., 2000; Destin et al., 2017), the present study also examined the whether the relationship between financial gambling motives and PG severity varied based on a measure of subjective SES, perceived deprivation. The findings regarding the association between perceived deprivation and PG in the present study are in line with prior works demonstrating an association between perceived deprivation and gambling urges (Callan et al., 2008, 2015) and PG (Mishra & Meadows, 2018; Tabri et al., 2015, 2017b). That is, the significant positive, direct effect of perceived deprivation on PG in the present study further substantiates perceptions of being relatively deprived as a robust predictor of PG.

Yet, the present work expanded on prior literature by examining perceived deprivation as a moderator in the relationship between financial gambling motives and PG severity. Past works had considered the possibility that financial motives for gambling might be a mediator between perceived relative deprivation and disordered gambling in a sample of community gamblers (Tabri et al., 2015). In contrast to that study, the present study alternatively operationalized financial motives as a variable predictor of PG and perceived deprivation as a moderator. In part, this decision was predicated on the substantial evidence for financial motives as a robust, direct predictor of PG severity, which is best demonstrated by a recent meta-analysis of 47 studies that indicated a reliable, moderate effect size (Tabri et al., 2022). Conceptually, it also makes more sense to operationalize financial motives as a predictor given that the gambling motives are, theoretically speaking, understood to be the underlying reason for or causal factor explaining gambling participation (Stewart & Zack, 2008). Yet, it also stands to reason that financial motives are not likely to be uniformly predictive of problematic outcomes. An individual gambling and hoping to win money but not feeling as if they *must* win to address some hardship or inequity is intuitively less likely to demonstrate problematic gambling behaviors than an individual gambling while feeling that they must win to address such things. Such a supposition is also evident from qualitative research demonstrating that gamblers were primarily motivated by financial motives, with perception of current financial circumstances being found to play an additional role (Lloyd et al., 2021).

The present study’s findings expand our understanding of the above conjecture by demonstrating that such relationships (i.e., the variable relationship between financial motives and PG depending on personal levels of perceived relative deprivation) are observable in population-level samples. Further, it is the first study to compare the influence of perceived deprivation, a subjective assessment of one’s economic status, and income, an objective indicator of one’s financial resources, in the relationship between financial gambling motives and PG severity.

### Implications

#### Theoretical Implications

The findings of the present work underscore the complexity of the relationship between financial resources, financial gambling motives, and gambling outcomes and demonstrates the importance of considering both objective and subjective SES when assessing risk of PG. Findings indicated that when examining gambling behavior more broadly, as opposed to separating analyses by gambling activity, annual household income may not be associated with higher PG severity among those who gamble for financial purposes. In contrast, findings indicated that perceptions of being worse off financially compared to others and not having what one deserves is associated with increased PG severity. As such, among those who are motivated to gamble for monetary reasons, problems may arise in response to upward socioeconomic comparisons. Such upward comparisons may motivate gambling as a means improving economic status to that of similar, more affluent peers. This finding is consistent with theories of how social comparison processes operate in the experience of perceived deprivation. Specifically, the finding that people tend to make social comparisons of affluence with others they perceive to be more similar than dissimilar and based on attributes surrounding their own financial status (Kim et al., 2018b). Such comparisons are most often upward (i.e., focused on those who are better off), then downward (Kim et al., 2018b). These findings have unique implications with regards to examinations of economic inequality and gambling outcomes, underscoring the importance of specificity with regards to how constructs may act differently in groups of varying SES. Findings have implications for the functional analysis of gambling problems, underscoring the importance of considering the role of monetary resources and how individuals evaluate or perceive them in gambling cognitions and behavior. Findings from the present study not only substantiate recommendations of paying attention to clients’ financial focus in the prevention and treatment of gambling problems (Lloyd et al., 2021; Tabri et al., 2017a), but also underscore a potential need to, in certain cases, look past objective financial standing, also examining feelings of dissatisfaction stemming from the belief that one is financially deprived in comparison to others. That is, though objective SES is indeed an important part of understanding why one is motivated to gambling and the likelihood of experiencing gambling related harms, subjective perceptions also matter a great deal, particularly as they related to self-reported symptoms of GD.

#### Clinical Implications

The present work may have implications with regards to the design of PG prevention and treatment strategies. The extant literature on gambling motives has clinical utility in its use as a means of subtyping people who gamble, often for the purpose of developing responsible gambling initiatives that aim to prevent gambling harm and connect people experiencing harm with resources for treatment (Dechant, 2014). For responsible gambling initiatives to be efficacious, initiatives must be able to accurately target factors in PG risk. The present study demonstrates that financial motives are linked to PG irrespective of objective financial standing. As such, if it is assumed that financial reasons do not play a role in gambling behavior based on objective financial standing alone, responsible gambling messaging could fail to reach subsets of at-risk gamblers. In cases in which financial motives are thought to be the reason for gambling, clinicians and responsible gambling initiatives might focus on making the odds of winning vs. losing clearer to individuals as a means of mitigating risk (Wohl et al., 2010). More to the point, responsible gambling initiatives should likely highlight the improbability of gambling leading to positive financial outcomes and acknowledge that financial motives may be considered a warning sign for PG, particularly among those that perceive themselves as economically disadvantaged.

Regarding treatment, findings are consistent with prior works underscoring the utility of motivation-matched approaches to treat PG (Stewart et al., 2011, 2016). For example, Stewart et al. (2011) developed and tested a gambling treatment program which personalizes treatment based on gambling motives that was demonstrated to be effective in reducing PG severity. However, this program did not consider financial motives. The present work highlights the importance of considering financial motives, as well as the conditional and cognitive factors associated with financial motives (e.g., perceived relative deprivation), in future attempts to develop motive-matched treatments.

While no gambling treatment approaches exist that aim to address perceived deprivation, researchers in the field have outlined relevant intervention points. For example, Callan et al. (2011) recommended the use of strategies aimed at reducing perceived deprivation to alleviate the risk of disordered gambling. In addition, Tabri et al. (2017a) points to the potential use of cognitive behavioral therapy techniques aimed at addressing gambler’s financial focus and decrease the degree of importance they attach to financial success in terms of their self-worth, supplementing it with other domains such as interpersonal relationships. Richard et al. (2017) discussed practical treatment considerations for addressing financial consequences of gambling among those who gamble to improve their SES. Within, the authors recommended Motivational Interviewing (MI), particularly for those lacking financial resources, due to MI focusing on helping individuals identify the motivations maintaining gambling while also increasing awareness of the person’s own reasons for wanting to stop gambling (Yakovenko et al., 2015).

### Limitations and Future Research

The present study has limitations that are worth noting. First, self-report measures were used, which have established limitations (Chan, 2009). In addition, the data used was cross-sectional and thus causal inferences cannot be drawn from findings. Research using a longitudinal design is needed to examine whether financial motives predict PG overtime and whether fluctuations in financial standing at differing time points impact the relationship. Longitudinal works are also needed to assess the relative stability of financial motives over time given research demonstrating that initial motives for gambling can differ from the motives that maintain PG later in life (Grubbs & Rosansky, 2020). In addition, the present study did not examine whether the relationships between key variables differed with regard to preferences in gambling activities, which is relevant given the evidence that SES influences preferences in terms of type of gambling. For example, research supports a unique relationship between low SES and lottery gambling (Ariyabuddhiphongs, 2011; Fu et al., 2021).

The present study solely examined financial motives, as opposed to other established motives for gambling (e.g., enhancement, coping, and social; Dechant, 2014; Stewart & Zack, 2008). It is worth noting that gambling motives are intercorrelated and thus may operate simultaneously. For example, individuals motivated to gamble for monetary reasons, and experience distress associated with gambling-related problems, would also be expected to gamble for negative affect alleviation. As such, attributing gambling behavior to a single motive may provide an incomplete interpretation of the motivations underlying gambling. Given this, future studies should examine financial motives combined with other gambling motives in examinations of PG. Further, the present study examined income, which is only one objective indicator of SES. The decision to focus solely on income was rooted in its focus on access to monetary resources, making it the best aspect for comparisons with the used measure of perceived deprivation, a subjective measure that is focused on evaluations of one’s resources in comparison to others. Still, income does not represent the full range of factors comprising SES and future works should examine other SES aspects (e.g., education and employment) given level of education and employment status have been found to also contribute to risk of PG (Ekholm et al., 2014). It may also be worth considering alternative measures of financial standing in future works, such as examining personal disposable or discretionary income, as opposed to household annual income. Further, there are limitations associated with the decision to drop items from the PRDS on the basis of internal consistency issues. Such internal consistency issues with the PRDS have been noted in past works (Callan et al., 2008; Kim et al., (2018a). In future studies, researchers are encouraged to use the modified version of Callan et al.’s (2008) PRDS, which modified the wording of items and added an additional item to improve the internal consistency (Callan et al., 2011).

Lastly, the present work used a census-matched, stratified, national sample instead of a sample comprised specifically of potentially vulnerable populations. Future studies should examine the relationship between these constructs in treatment seeking samples and in vulnerable populations, particularly those of lower income for whom gambling might be seen as a viable means of increasing socioeconomic standing. Research of this sort is particularly needed among minoritized populations, who appear to be at increased risk of PG and experience quicker monetary consequences as a product of existing economic disparities (Richard et al., 2017), which are likely to increase monetary motives for gambling.

## Conclusion

The present study aimed to examine the relationship between financial motives for gambling and PG based on income and perceived deprivation. Data from 1,348 gamblers were analyzed. Results confirmed findings in prior literature regarding financial gambling motives and perceived deprivation as robust predictors of PG. The findings regarding the interaction of financial motives and income were inconsistent with expectations, with no significant interaction being found. However, consistent with expectations, the strength of the association between risk of financial motives and higher levels of PG was found to increase at higher levels of perceived deprivation. Such findings suggest that subjective evaluations of one’s financial state in comparison to others may play a role in the relationship between being motivated to gamble for financial purposes and PG. In conclusion, our findings demonstrate the importance of considering how differences in both objective and subjective SES may contribute to risk of PG among those who gamble for financial reasons.

## Data Availability

The data and supplementary materials are available from the open science framework database (link: https://osf.io/9xbv8/.)
